# Bear-Baiting May Exacerbate Wolf-Hunting Dog Conflict

**DOI:** 10.1371/journal.pone.0061708

**Published:** 2013-04-17

**Authors:** Joseph K. Bump, Chelsea M. Murawski, Linda M. Kartano, Dean E. Beyer, Brian J. Roell

**Affiliations:** 1 School of Forest Resources and Environmental Science, Michigan Technological University, Houghton, Michigan, United States of America; 2 Wildlife Division, Michigan Department of Natural Resources, Marquette, Michigan, United States of America; 3 Biological and Environmental Sciences, University of Helsinki, Helsinki, Finland; Université de Sherbrooke, Canada

## Abstract

**Background:**

The influence of policy on the incidence of human-wildlife conflict can be complex and not entirely anticipated. Policies for managing bear hunter success and depredation on hunting dogs by wolves represent an important case because with increasing wolves, depredations are expected to increase. This case is challenging because compensation for wolf depredation on hunting dogs as compared to livestock is less common and more likely to be opposed. Therefore, actions that minimize the likelihood of such conflicts are a conservation need.

**Methodology/Principal Findings:**

We used data from two US states with similar wolf populations but markedly different wolf/hunting dog depredation patterns to examine the influence of bear hunting regulations, bear hunter to wolf ratios, hunter method, and hunter effort on wolf depredation trends. Results indicated that the ratio of bear hunting permits sold per wolf, and hunter method are important factors affecting wolf depredation trends in the Upper Great Lakes region, but strong differences exist between Michigan and Wisconsin related in part to the timing and duration of bear-baiting (i.e., free feeding). The probability that a wolf depredated a bear-hunting dog increases with the duration of bear-baiting, resulting in a relative risk of depredation 2.12–7.22× greater in Wisconsin than Michigan. The net effect of compensation for hunting dog depredation in Wisconsin may also contribute to the difference between states.

**Conclusions/Significance:**

These results identified a potential tradeoff between bear hunting success and wolf/bear-hunting dog conflict. These results indicate that management options to minimize conflict exist, such as adjusting baiting regulations. If reducing depredations is an important goal, this analysis indicates that actions aside from (or in addition to) reducing wolf abundance might achieve that goal. This study also stresses the need to better understand the relationship among baiting practices, the effect of compensation on hunter behavior, and depredation occurrence.

## Introduction

Depredation (i.e., the killing or injuring of domestic animals by wildlife) is a human-wildlife conflict often addressed in conservation policy, and stakeholder support for such policy is influenced by actions taken or not taken in response to depredation [1], [2]. Mitigating depredation of bear-hunting dogs (*Canis lupus familiaris*; hereafter dogs) by gray wolves (*Canis lupus*; hereafter wolves;) is especially challenging [3]–[13]. While compensation programs developed in response to wolf depredation of livestock are widespread and generally supported, compensation for wolf depredation on dogs is less common and more likely to be opposed [11], [14]–[16], [17]. In the case of dogs then, actions that minimize the likelihood of depredation events are a premium option for stakeholders and a continued need for conservation science [12]. This need is expected to grow because wolves are present in or likely to recolonize many of the states that permit bear-baiting. Currently, 30 US states permit black bear hunting, 17 allow hunting with dogs, and 10 also allow the use of bait as a hunting method. Self-sustaining wolf populations are currently found in eight states: Alaska, Arizona, Idaho, Minnesota, Montana, Wisconsin, and Wyoming.

Minimizing depredations is especially important in the Great Lakes region of the United States where the US Fish and Wildlife Service recently removed wolves from the federal endangered species list and wolf management is now under state and tribal authority [18]. Within this region wolf-dog conflict most frequently occurs with dogs used to hunt black bear (*Ursus americanus*) [3], [15], [19], [20]. Dogs used to hunt black bear are more commonly involved in wolf depredation events than dogs considered house pets [20], and are the most costly indemnification category among domestic animals in Wisconsin [21]. The greater loss of dogs used to hunt black bear may reflect the increased likelihood for these animals to encounter wolves while humans are not immediately present during hunting or training activities [19], [20].

While wolf-dog conflict is a regional issue, temporal trends of such depredations differ markedly between states. As wolf abundance has increased, the number of hunting dog depredation events was also expected to increase due to increased chance encounters [3, 15, 19. 22]. Data collected during 2002–2008 in Wisconsin support this expectation, but data collected during 1996–2009 in Michigan do not [20]; to our knowledge an analysis of earlier depredation data has not been completed. The apparent difference in depredations between states exists despite similarities in wolf populations, allowable hunting methods and rules, and habitat conditions for bears and wolves [23]–[27]. Michigan and Wisconsin are the only two US states within the Great Lakes region that permit bear hunting with bait, permit the use of hounds with or without bait, and contain wolf populations. Given these similarities, it is not clear why Wisconsin and Michigan exhibit markedly different wolf/bear-hunting dog depredation patterns, although researchers have proposed a number of untested explanations [19]. Here we test explanatory mechanisms related to bear-baiting (i.e., free feeding; hereafter baiting), hunting and baiting regulations, wolf-dog encounter rates, and hunter method and effort. Dissimilar compensation policies between Wisconsin and Michigan may also contribute to different dog depredation patterns. To our knowledge, data to assess the net effect of compensation or lack thereof are unavailable, hence we discuss the potential implications of dissimilar compensation policies. The aim of this analysis and discussion is to identify potential options to improve wolf management and conservation by mitigating depredations.

## Methods

### Hypotheses

Encounter rates between wolves and their prey are ratio-dependant in similar systems [28], which suggests a parallel expectation for encounters with dogs. We examined the ratio of bear-hunting permits sold per wolf in each state to determine if the proportion of bear hunters relative to wolves is associated with differences in depredation rates. If encounter rates contribute to observed differences in depredation trends, we expect that the number of bear-hunting permits sold per wolf to be significantly higher in Wisconsin than in Michigan.

Dissimilar timing and duration of baiting between MI and WI may also explain the observed difference in wolf depredation on dogs. Wisconsin allows baiting to begin much earlier in the year (∼mid-April) than Michigan (∼mid-August). Both states allow training of dogs to begin in early July annually. Thus, Wisconsin has a baiting period of ∼2.5 months before dog training is allowed that does not exist in Michigan and baiting in Michigan begins ∼4 months later than in Wisconsin. Because wolves tend to be strongly territorial, routinely kill canids [trespassing wolves, coyotes (*Canis latrans*) and dogs] in defense of their territories [29], [30], and are readily attracted to bait sites [19], it is thought that the extended, pre-training baiting period in Wisconsin provides wolves increased opportunity to discover and defend bait sites [19], [20]. Indeed, this mechanism has been previously proposed based on observations of wolves using bait sites for food as documented by cameras, tracks, and the stomach contents of a captured wolf [19]. Bear hunters using dogs frequently (∼90% of the time) start their dogs at bait sites [31] and it seems reasonable to expect that dogs in Wisconsin would be more likely to encounter wolves that are in the proximity of and potentially defensive of bait sites [19]. A greater percentage of bear-hunters using dogs and/or bait methods in Wisconsin versus Michigan could also contribute to the observed difference in depredations patterns. Similarly, hunter effort is a potential explanatory factor if greater in Wisconsin than Michigan and we test for such differences.

If the duration of baiting is important in allowing wolves to associate with and potentially defend bait sites, we predict the likelihood of wolf depredation on dogs would be greatest at the first opportunity for an encounter between the two species, which is at the time when training with bait begins in both states. We expect this because prior to training with bait, wolves can freely visit bait sites without encountering dogs. It is reasonable to expect that the longer free association with bait sites is possible, then a greater potential exists for wolves to be present in the vicinity of bait sites once training begins. This could result in a greater likelihood of conflict associated with bait sites [19]. Consequently, we expect that the chance of depredation would be higher in Wisconsin than in Michigan given the increased duration of bear baiting.

### The Data

We analyzed records of verified wolf depredation on dogs provided by the Michigan Department of Natural Resources (MDNR) and the WDNR since the first dog depredation record in each state through 2010. Wolves recolonized Wisconsin in ∼1979 [32] and Michigan in ∼1989; the first dog depredation records occurred in Wisconsin in 1986 or 1987 [19], [32] and in Michigan in 1996. Depredation events consisted of ≥1 dog killed or injured on a single occasion with the cause attributed to wolves by MDNR, WDNR, or U.S. Department of Agriculture, Wildlife Services personnel. We obtained annual wolf abundance, number of bear-hunting permits, hunter method (percentage bait only, dogs only, dogs & bait, other), and hunter effort (days afield) data from MDNR and WDNR for areas in which bear hunting with dogs is permitted. All bear management units within Michigan’s Upper Peninsula allow bear hunting with dogs [33] and Wisconsin permits bear hunting with dogs in three of the state’s four bear hunting zones [34]. Hence, the annual sampling unit is the area (unit or zone) in which bears may be hunted with dogs in each state. Current and recent wolf distribution in each state overlaps these areas.

### Statistical Analyses

To assess the relationship between counts of depredations on dogs and explanatory factors between states we used generalized linear models (GLM) with a logarithmic link function and a Poisson error distribution because high annual counts of depredations can be rare events [35], [36]. Candidate explanatory factors included state, wolf abundance, the ratio of bear hunting permits sold per wolf as an encounter factor, hunter method (dogs only, bait only, dogs & bait, other, total dogs, total bait), and hunter effort (i.e., days afield). We first tested for parallelism (i.e., no difference) of explanatory effects between states by assessing the significance of state×explanatory factor interactions terms. If explanatory factor effects did not differ between states, interactions terms were not included in subsequent models. The annual number of bear permits sold was also included as an offset variable to adjust for possible differences in the number of hunters and to compare total bear hunter exposure between states. Unless otherwise noted, examination of Spearman’s correlations (nonparametric) and variance inflation factors did not reveal multicollinearity issues among explanatory variables. We tested for overdisperson using Pearson χ^2^/DF and if present, a variance inflation factor correction was applied [36].

Model inferences were made using Akaike Information Criterion corrected for small sample size (AIC_C_) [37]. Specifically, AIC_C_ was calculated for models with all possible combinations of explanatory factors and interaction terms with state. We used ΔAIC_C_ and AIC weights (W) to indicate our degree of confidence in each model relative to other potential models; models with ΔAIC_C_ <2 are considered worthy as explanatory [37]. Unless otherwise mentioned, none of the models described suffered from high levels of autocorrelated residuals, nor did inspected residual plots reveal obvious indications of heteroscedasticity or excessive influence of individual observations. We conducted all analyses using JMP (version 9.0.2, SAS Institute Inc.).

To answer the question “Is the likelihood of depredation associated with the duration of baiting?” we examined whether or not a depredation event occurred (*Y*, dependent variable), as related to the time (*n = *123 days) since the beginning of training with bait (*X_1_*), in each state (*X_2_*). The time period for this analysis encompassed the bear training and hunting seasons in each state (July-November). To do so we used a two-predictor logistic regression model with logit link function and a binomial error distribution [38]:



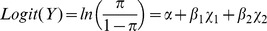



Therefore the relative probability of a depredation event occurring (*P*) is:
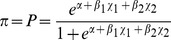
(2)where *α* is the *Y* intercept. Coefficients were estimated by maximum likelihood.

## Results

**Figure 1:**
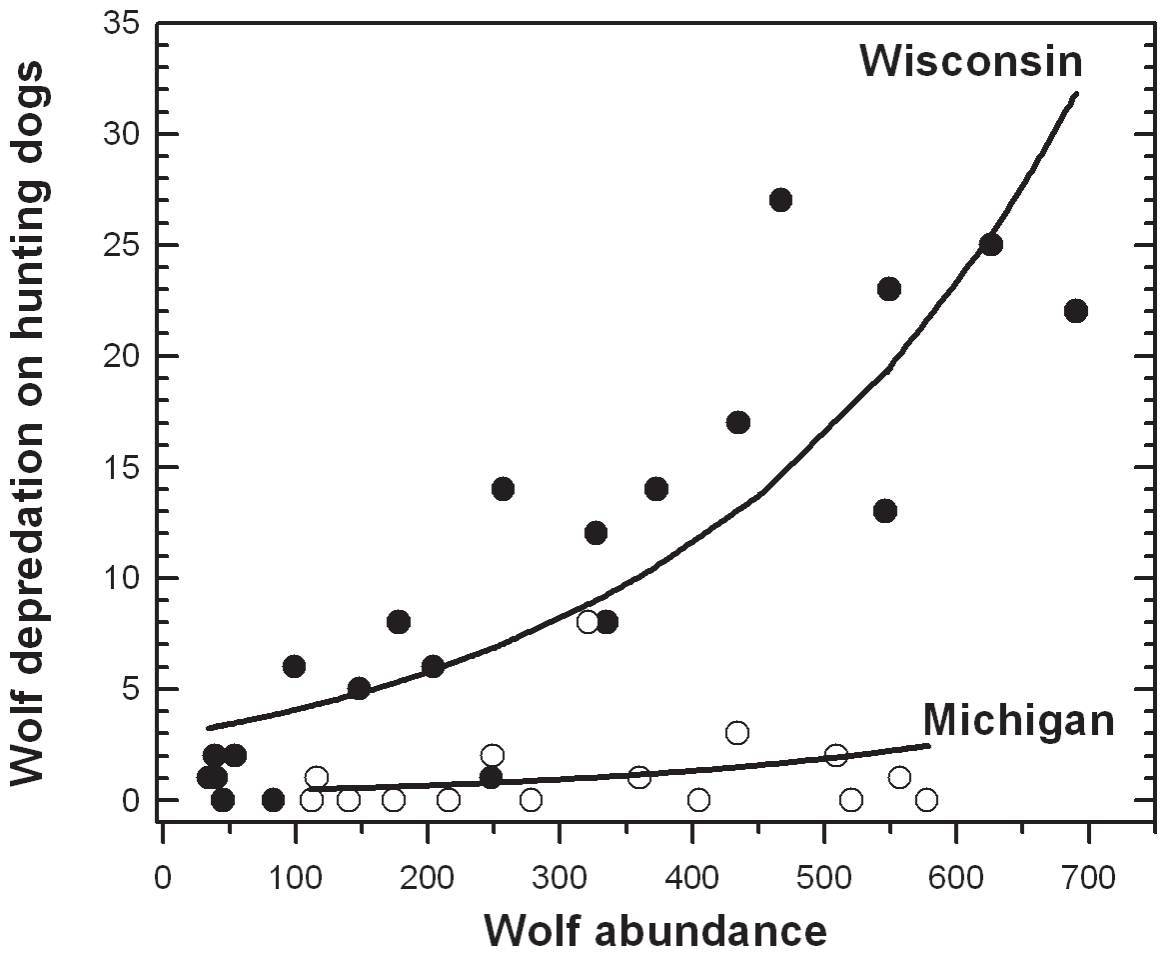
Distinct wolf conflict patterns in the upper Great Lakes region, USA. Poisson log-linear relationship between annual totals of wolf depredation (i.e. kill or injury) on bear-hunting dogs (*y-axis*) and annual estimates of wolf abundance (*x-axis*) in Wisconsin (1990–2010; *closed symbols*) and Michigan (1996–2010; *open symbols*).

Our interaction tests for parallelism indicated the following candidate explanatory factors did not differ between states and hence do not likely explain differences in depredations (Fig. 1): wolf abundance (*p = *0.337), the ratio of bear hunting permits sold per wolf, i.e. the encounter factor (*p = *0.594), hunter method [dogs only (*p = *0.649), bait only (*p = *0.262), dogs & bait (*p = *0.336), other (*p = *0.211), dogs total (*p = *0.171), bait total (*p = *0.901), and hunter effort, i.e. days afield (*p = *0.223). State is the only significant (*p*<1×10^−4^) factor present in all of the models that best explained trends in annual totals of wolf depredation on dogs between Michigan and Wisconsin (Table 1). This result indicates that depredation counts are significantly different between states, i.e. higher in Wisconsin, and confirms earlier findings that examined data from fewer years [19], [20]. The mean annual dog depredation count for Wisconsin (1986–2010) is 8.6, median = 6, range 0–27; for Michigan (1996–2010) the mean = 1.2, median = 0, and the range = 0–8. In two of the top five models the ratio of bear permits sold per wolf (i.e., encounter factor) is significant (*p = *0.028 and *p = *0.032) and hunters using bait only and any method involving dogs appear significant (*p = *0.018 and *p = *0.016, respectively) in separate models (Table 1). Hunter effort (i.e., days afield) only appears in the weakest of top models, but is not significant (Table 1). Use of the annual number of bear permits sold as an offset variable to adjust for possible differences in the number of hunters did not change these results, indicating comparable total bear hunter exposure between states.

**Table 1:** Comparison of best performing models explaining trends in wolf depredation on bear-hunting dogs in the Wisconsin and Michigan, USA, by Akaike’s information criterion & weight.

Explanatory factors*	AIC_C_ a	ΔAIC_C_ b	W^c^
Wolves (0.531), State (<0.001),Bait total (0.28)	36.30	0.00	0.30
State (<0.0001), Bait only (0.018)	36.77	0.47	0.24
State (<0.0001), Dogs total (0.016)	37.16	0.86	0.20
State (<0.0001), Encounter (0.028),Bait total (0.948)	37.74	1.44	0.15
State (<0.0001), Days afield (0.588),Encounter (0.032)	38.17	1.87	0.12

* Encounter = the ratio of bear hunting permits sold per wolf (see methods). Numbers in parenthesis under explanatory factors are *p*-values for the five best-performing models.

a AIC_C_ is Akaike’s information criterion, corrected for small sample size.

b ΔAIC_C_ is AIC_C_ for the model of interest minus the smallest AIC_C_ for the set of models being considered. We only considered models with ΔAIC_C_ ≤2.

c W is the Akaike’s weight of each model. The ratio of one model’s weight to another estimates how many times more support the data provide for that model over the other.

**Figure 2:**
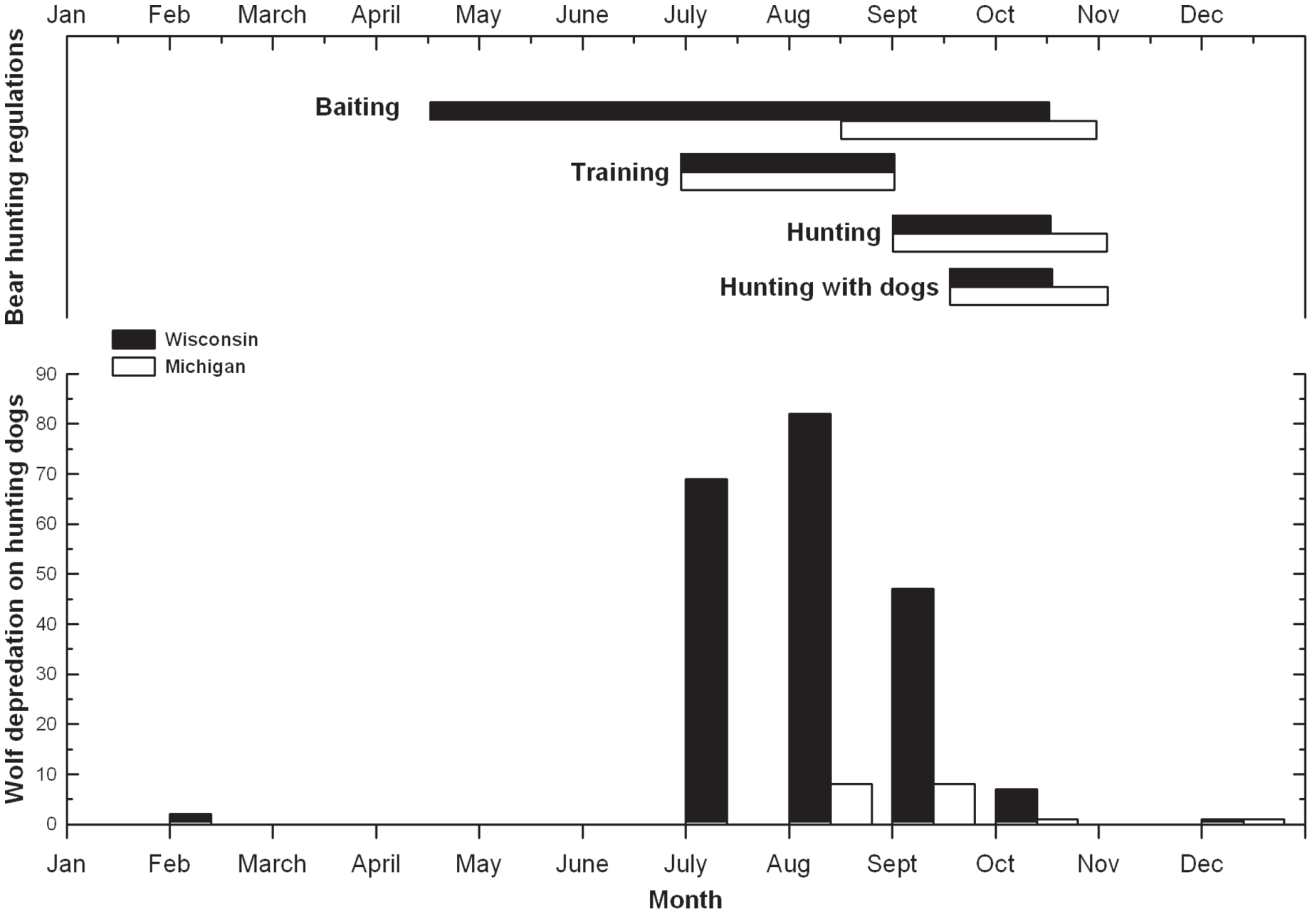
Wolf conflict timing and bear hunting in the upper Great Lakes region, USA. *Lower panel*: cumulative wolf depredations on bear-hunting dogs (*y-axis*) each month (*x-axis*) from 1980–2010 in Wisconsin (*closed bars*) and Michigan (*open bars*). *Upper panel*: General timing of bear-baiting, training, and hunting regulations (*y-axis*) in each state; *x-axis* and bar symbols are the same as in lower panel. In Wisconsin there is a pre-training baiting period of ∼2.5 months that does not exist in Michigan and baiting in Michigan begins ∼4 months later than in Wisconsin.

The peak occurrence of hunting dog depredations coincides with training or hunting with bait in each state, beginning in July in Wisconsin and August in Michigan (Fig. 2). While the annual timing of the increase is similar between the states, Wisconsin depredations rates are up to 5×, greater (e.g. in August) than in Michigan (Fig. 2). Logistic regression analysis indicates that the probability of depredation is greater in Wisconsin (i.e., coefficient for state = 1.27; *p*<1×10^−4^), and the probability of depredation decreases with time since training with bait (i.e., coefficient for time = −0.0241; *p*<1×10^−4^; Table 2). Firth-biased adjusted maximum likelihood estimates for small sample size yielded equivalent results. The goodness-of-fit test yielded a χ^2^ (*df* 243) of 124.6 and was insignificant (*p = *0.3785), which indicated that the whole model fit the data well (Table 2).

**Table 2:** Logistic regression analysis of the probability of wolf depredation on hunting dogs in relation to time since training with bait in Wisconsin and Michigan, USA.

Predictor	β	*SE* β	Wald’s χ^2^	*df*	*p*	*e^β^* (odds ratio)
Constant	0.8119	0.3115	4.79	1	0.091	NA
Time since training with bait	−0.0241	0.0048	25.11	1	<0.0001	0.9762
State	1.2732	0.1696	44.16	1	<0.0001	3.5723
Test			χ^2^	*df*	*p*	
Overall model evaluation						
Likelihood ratio test			39.01	1	<0.0001	
Wald test			44.16	1	<0.0001	
Goodness-of-fit test						
Likelihood ratio test			249.21	243	0.3785	

*Note*. NA = not applicable.

**Figure 3:**
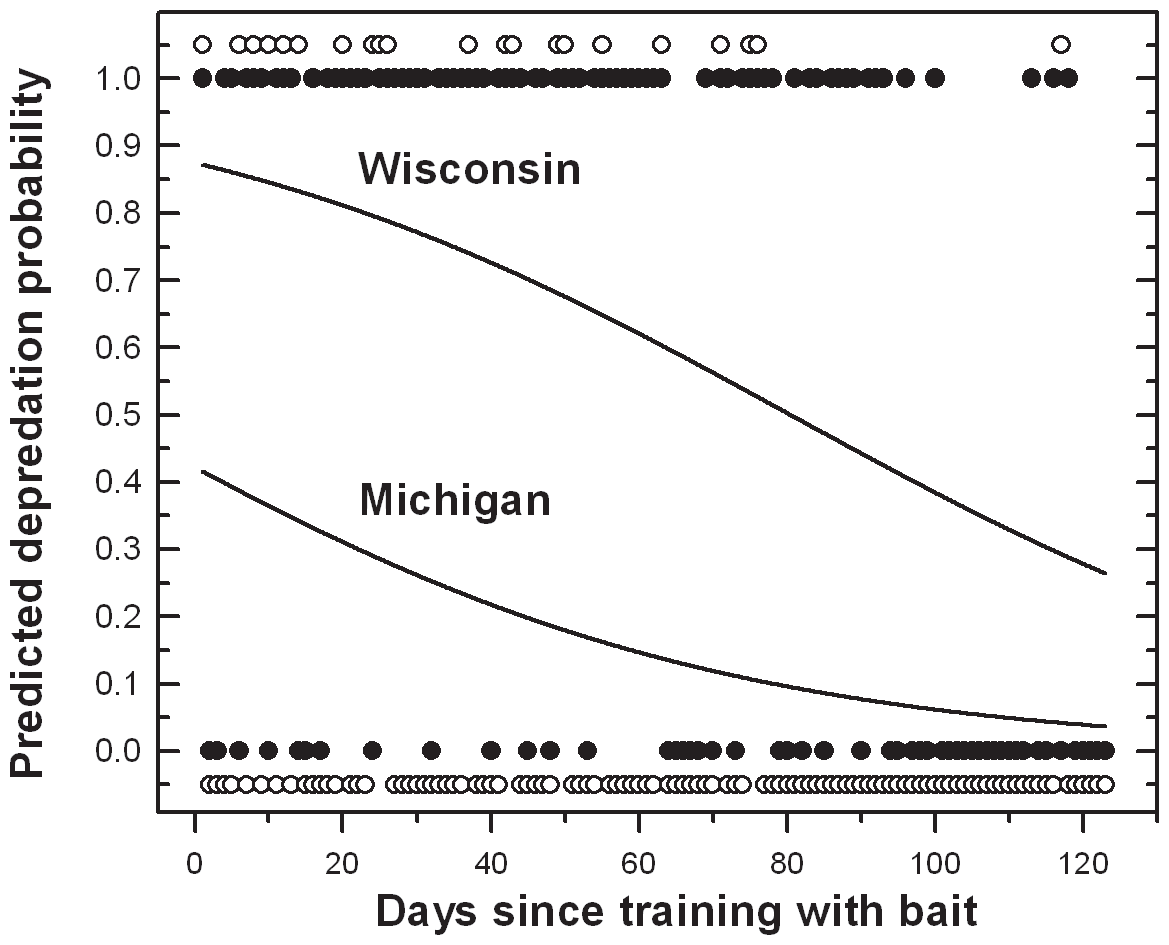
Wolf conflict and the duration of bear-baiting in the upper Great Lakes region, USA. Predicted probability of a wolf depredation on bear-hunting dogs (*y-axis*) versus the number of days since training with bait began (*x-axis*) in Wisconsin (*upper line*) and Michigan (*lower line*). Each point represents a day since training with bait began in Wisconsin (*closed symbols*) and Michigan (*open symbols*). Note that open symbols for Michigan are offset from (0) and (1) probability so as to not overlap symbols for Wisconsin. The odds of a depredation event occurring in Wisconsin were 3.57× greater than the odds in Michigan; a relative depredation risk 2.12–7.22× greater in Wisconsin.

According to this logistic model, the probability of a depredation event occurring decreases since training with bait (*p*<1×10^−4^) and by state (*p*<1×10^−4^). The closer to the first date of training with bait (i.e., ∼July 1^st^ in Wisconsin and ∼August 15^th^ in Michigan), the more likely it was that a depredation event occurred. For each additional day since training with bait, the odds of a depredation event occurring decreased from 1.0 to 0.976 ( = *e* ^−0.0241^; Table 2). Given the same time since training with bait began, the odds of a depredation event occurring in Wisconsin were 3.57× ( = *e* ^1.273^; Table 2) greater than the odds in Michigan (Table 1; Fig. 3). For example, as time increases from the first day of training with bait until the end of hunting with dogs (i.e., ∼123 days), the probability decreases from 0.87 to 0.26 in Wisconsin and from 0.41 to 0.36 in Michigan, indicating a relative risk 2.12–7.2× greater in Wisconsin.

## Discussion

Collectively, these models indicated the ratio of bear hunting permits sold per wolf, and hunter method are important factors affecting regional wolf depredation trends in the Upper Great Lakes region of the US, but strong differences exist between Michigan and Wisconsin (Fig. 1). The strong difference in wolf/bear-hunting dog depredation trends between Michigan and Wisconsin (Fig. 1) is related, at least in part, to distinct baiting regulations (Fig. 2). Model results (Table 2) support the hypothesis that the likelihood of a wolf depredation on dogs decreases with time from the beginning of training with bait (Fig. 3). Potential differences in wolf abundance, wolf-dog encounter rate, hunter method and effort between states, which could also explain distinct state differences, were not detected (Results & Table 1). Moreover when considering wolf-dog encounter rates, the area of Michigan’s Upper Peninsula (∼42,035 km^2^, excluding Isle Royale National Park) is less than the area of the three WDNR bear management zones *A*, *B*, and *D* (∼47,243 km^2^), in which bear hunting with dogs is permitted. With a similar ratio of bear hunting permits sold per wolf, a smaller geographic area should increase encounter rates and exacerbate hunting-dog depredation conflicts, yet our results do not support this prediction. Given similar bear and wolf habitat [23]–[26] geographic area, and equivalent proportions of hunters using the same technique and spending similar time afield in each state, it is less likely that wolf depredation on hunting dogs is strongly related to landscape-level, random encounters. Our results better support the conclusion that the likelihood of wolf depredation on hunting dogs is likely linked to non-random encounters associated with baiting for bear and attracting wolves to focal points, i.e. bait piles.

While the duration and timing of baiting may exacerbate wolf-bear hunting dog conflict, other differences in baiting regulations between Michigan and Wisconsin exist. These differences may be contributing factors. Wisconsin limits bear-bait in volume and type; it is illegal to place >10 gallons (37.85 liters) of bait or use animal parts or animal by-products [34]. In contrast, Michigan permits baiting with animal parts and by-products, and bakery/confectionary products without quantity restrictions, but limits grain and vegetable products to two gallons (7.57 liters) [34]. It seems reasonable to expect that wolves, as carnivores, are more likely to locate, visit, and potentially defend unregulated quantities of animal parts or by-products than limited volumes of non-animal products as allowed in Michigan, but not Wisconsin. In comparing bait volume and type regulations, we would expect higher depredation rates in Michigan versus Wisconsin but that was not the case. Consequently, of the differences in baiting regulations between Michigan and Wisconsin (Fig. 2), the duration and timing of baiting appears to contribute to the observed difference in wolf depredation on dogs between the states (Fig. 1). If correct, this suggests that there are additional opportunities to mitigate wolf depredations on dogs (e.g. adjusting baiting regulations).

Dissimilar compensation policies between the states may also contribute to the difference in hunting dog depredation rates. More specifically, hunters in Wisconsin may be more likely to report losses because Wisconsin compensates dog owners for lethal wolf depredation (up to $2,500 USD) and will reimburse veterinary costs for non-lethal attacks, while Michigan does neither [20]. However, the authors have no reason to suspect underreporting in Michigan as depredation events are frequently publicized (e.g. Michigan Sportsman, Upper Michigan Source), Michigan citizens regularly ask why Michigan does not compensate for dog depredation as in Wisconsin, the MDNR works closely with bear and hunting dog groups, both of which consistently report depredations, and an indemnification policy to compensate for wolf depredations on dogs is currently under consideration in Michigan. Even if annual depredation counts in Michigan are increased 5×, the count trend is significantly less than in Wisconsin (*p = *0.0086; same GLM methods described previously), which indicates that possible low to moderate underreporting in Michigan does not change our results. Given publicity associated with depredation events and the shared interest by hunting dog groups and the MDNR to minimize depredations, it is unlikely that Michigan depredations are underreported by 500%.

In addition to a reporting inducement, compensation also creates an incentive for riskier behavior [39], such as knowingly releasing dogs in areas identified as dog depredation zones and wolf caution areas, which Wisconsin identifies and disseminates in near real-time. The net effect of compensation on reporting and risk-taking behavior is not well understood and to our knowledge, data to assess the net effect are not available. We do not maintain that compensation effects are unimportant or absent from this system. Rather, we maintain that empirical evidence to understand compensation effects is lacking and compensation can potentially create multiple and sometimes opposing effects [40]. Untested compensation effects are therefore not necessarily the most parsimonious explanatory factor a priori. Insofar as this study identifies baiting as a factor that likely effects wolf depredation on hunting dogs, it also identifies factors for which data are lacking.

Hunters use baiting to increase their success and bear hunter success in Wisconsin (∼56%) [31] is nearly double that of Michigan (∼29%) on average over the past two decades. If the timing and duration of baiting contribute to the observed differences in wolf depredations on hunting dogs and bear hunting success regionally, then these results have identified a potential tradeoff between high bear hunting success and increased wolf/bear-hunting dog conflict. This tradeoff illustrates a general problem in conservation and wildlife management: practices sometimes permitted to increase the likelihood of harvest worsen human-wildlife conflict. To our knowledge, previous studies have not identified that wildlife baiting might exacerbate depredation rates. Hence, our results underscore the need to examine the relationship among baiting practices, depredations, and compensation programs. This relationship likely varies across scales and while the regional results that we report identify a general issue, we do not doubt that important variation may exist at smaller scales and across seasons. For example, smaller-scale analyses could identify important heterogeneity in the risk of dog depredation and help develop regulations to minimize bait site visitation by non-target species.

Minimizing wolf depredations on all types of hunting dogs has important economic and social impacts. For example, even though compensation for wolf depredation on hunting dogs is more likely to be opposed than compensation for livestock [11] and livestock depredation events are more frequent [11], [15], [20] compensation for wolf depredation on hunting dogs can be more expensive for state agencies. From 1985–2010 in Wisconsin, wolf damage funds paid for losses of sheep, calf, and cattle and vet fees for injured cattle (*n* = 492) totaled $319,652 USD, while funds paid for killed hunting dogs and hunting dog vet fees (*n* = 208) were $418,102 USD [39]. Missing calves attributed to wolf depredation (*n* = 157) over the same period cost $129,229 USD [41]. Continued compensation for hunting dog depredations, especially if depredations continue at their current rate in Wisconsin (Fig. 1), is arguably unsustainable economically and socially [11]. Indeed, following federal delisting in December 2011, WDNR announced their intent to reduce statewide wolf numbers from ∼690–750 to ∼350–375, with the hope of decreasing wolf depredations. If a reduction in depredations is the goal, actions aside from (or in addition to) reducing wolf abundance might achieve that goal.
